# Neuroimaging and plasma evidence of early white matter loss in Parkinson’s disease with poor outcomes

**DOI:** 10.1093/braincomms/fcae130

**Published:** 2024-04-16

**Authors:** Angeliki Zarkali, Naomi Hannaway, Peter McColgan, Amanda J Heslegrave, Elena Veleva, Rhiannon Laban, Henrik Zetterberg, Andrew J Lees, Nick C Fox, Rimona S Weil

**Affiliations:** Dementia Research Centre, Institute of Neurology, University College London, London WC1N 3AR, UK; Dementia Research Centre, Institute of Neurology, University College London, London WC1N 3AR, UK; Huntington’s Disease Centre, Institute of Neurology, University College London, London WC1B 5EH, UK; UK DRI Fluid Biomarker Lab and Biomarker Factory, University College London, London WC1E 6BT, UK; UK DRI Fluid Biomarker Lab and Biomarker Factory, University College London, London WC1E 6BT, UK; UK DRI Fluid Biomarker Lab and Biomarker Factory, University College London, London WC1E 6BT, UK; Dementia Research Centre, Institute of Neurology, University College London, London WC1N 3AR, UK; UK DRI Fluid Biomarker Lab and Biomarker Factory, University College London, London WC1E 6BT, UK; Reta Lila Weston Institute of Neurological Studies, University College London, London WC1N 1PJ, UK; Dementia Research Centre, Institute of Neurology, University College London, London WC1N 3AR, UK; National Hospital for Neurology and Neurosurgery, University College London Hospitals, London WC1N 3BG, UK; Dementia Research Centre, Institute of Neurology, University College London, London WC1N 3AR, UK; National Hospital for Neurology and Neurosurgery, University College London Hospitals, London WC1N 3BG, UK; Movement Disorders Centre, University College London, London WC1N 3BG, UK; The Wellcome Centre for Human Neuroimaging, Institute of Neurology, University College London, London WC1N 3AR, UK

**Keywords:** Parkinson’s disease, poor outcome, neuroimaging, plasma biomarkers, white matter

## Abstract

Parkinson’s disease is a common and debilitating neurodegenerative disorder, with over half of patients progressing to postural instability, dementia or death within 10 years of diagnosis. However, the onset and rate of progression to poor outcomes is highly variable, underpinned by heterogeneity in underlying pathological processes. Quantitative and sensitive measures predicting poor outcomes will be critical for targeted treatment, but most studies to date have been limited to a single modality or assessed patients with established cognitive impairment. Here, we used multimodal neuroimaging and plasma measures in 98 patients with Parkinson’s disease and 28 age-matched controls followed up over 3 years. We examined: grey matter (cortical thickness and subcortical volume), white matter (fibre cross-section, a measure of macrostructure; and fibre density, a measure of microstructure) at whole-brain and tract level; structural and functional connectivity; and plasma levels of neurofilament light chain and phosphorylated tau 181. We evaluated relationships with subsequent poor outcomes, defined as development of mild cognitive impairment, dementia, frailty or death at any time during follow-up, in people with Parkinson’s disease. We show that extensive white matter macrostructural changes are already evident at baseline assessment in people with Parkinson’s disease who progress to poor outcomes (*n* = 31): with up to 19% reduction in fibre cross-section in multiple tracts, and a subnetwork of reduced structural connectivity strength, particularly involving connections between right frontoparietal and left frontal, right frontoparietal and left parietal and right temporo-occipital and left parietal modules. In contrast, grey matter volumes and functional connectivity were preserved in people with Parkinson’s disease with poor outcomes. Neurofilament light chain, but not phosphorylated tau 181 levels were increased in people with Parkinson’s disease with poor outcomes, and correlated with white matter loss. These findings suggest that imaging sensitive to white matter macrostructure and plasma neurofilament light chain may be useful early markers of poor outcomes in Parkinson’s disease. As new targeted treatments for neurodegenerative disease are emerging, these measures show important potential to aid patient selection for treatment and improve stratification for clinical trials.

## Teaser

Zarkali *et al*. compare baseline multimodal imaging and plasma markers in patients with Parkinson’s who develop poor outcomes over 3 years. They show extensive macrostructural white matter loss and increased plasma neurofilament light with preserved grey matter and functional connectivity, highlighting the role of early white matter loss in Parkinson’s.

## Graphical Abstract

**Graphical Abstract:**
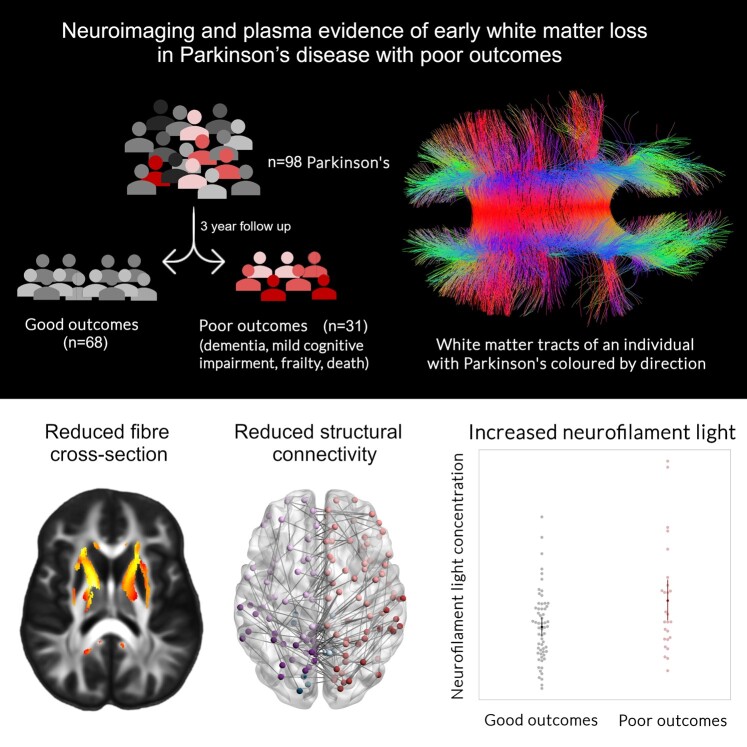

## Introduction

Parkinson’s disease (PD) is the second commonest neurodegenerative condition.^1^ As well as the well-described motor symptoms of rest tremor, rigidity and bradykinesia, around half of all patients will develop dementia within 10 years’ of diagnosis,^2^ with Parkinson’s disease dementia having higher societal and economic burden than other dementias.^3,4^ Other poor outcomes include frailty and falls due to postural instability. However, the timing and rate of clinical deterioration vary greatly^2,5^ as does the underlying brain pathology, with varying degrees and locations of alpha-synuclein-containing Lewy-related pathology, as well as extent and severity of beta-amyloid and tau pathological accumulations.^6^ Although factors such as older age, male sex and baseline cognition, particularly visuoperceptual dysfunction,^7^ are associated with poor clinical outcomes, and clinical algorithms to predict risk are being developed,^8,9^ the underlying changes in brain structure and function in patients at increased risk of PD dementia remain unclear.

Most studies examining neuroimaging changes associated with poor outcomes in PD have been limited to a single modality, mainly focusing on measures of grey matter, and results have been highly inconsistent.^10^ Both frontal^11^ and temporoparietal cortical thickness changes^12^ have been reported in people with PD who subsequently develop dementia, with several other areas implicated.^13,14^ Additionally, reduced volume of the cholinergic nucleus basalis of Meynert is linked to subsequent worsening cognition.^15,16^

However, grey matter change reflects neuronal loss,^17^ and evidence from animal models shows that white matter degeneration precedes neuronal loss in PD,^18,19^ suggesting that *in vivo* markers of white matter integrity, rather than grey matter, might be more sensitive to clinical severity in PD. Chung *et al.* recently showed white matter alterations in people with PD with mild cognitive impairment (PD-MCI) who progressed to develop dementia. Several tracts were implicated, including the arcuate fasciculus bilaterally and the left cingulum.^20^ However, that study evaluated patients with established cognitive impairment, and used only diffusion tensor imaging analysis that cannot accurately model fibres with divergent orientations, which make up the majority of fibre tracts in the brain.^21^

Instead, higher order diffusion models provide more accurate measures of crossing fibres, allowing better estimation of white matter *in vivo*. Fixel-based analysis is one such model, and was applied by Rau *et al*.^22^ to reveal reduction in fibre cross-section within the anterior body of the corpus callosum in people with PD with more severe disease. We recently applied fixel-based analysis to reveal widespread changes in people with PD with poor visuoperceptual function, who are at higher risk of dementia.^23^ Changes at the whole-network level may also provide more sensitive measures of poor outcome in PD: reduction in structural connectivity is seen in people with PD-MCI who subsequently progress to dementia;^20^ whilst long-range interhemispheric connections are more affected in people with PD at higher risk of dementia.^24^

Complementary to neuroimaging approaches, plasma and CSF measures are becoming more readily available, and provide insights into underlying processes, and as potential markers of severity. Neurofilament light chain (NFL) reflects axonal damage and shows higher CSF concentrations relating to white matter lesions in conditions including multiple sclerosis and Alzheimer’s.^25,26^ CSF NFL is higher in people with PD with established cognitive impairment,^27^ and plasma NFL is increased in people with PD who later developed PD-MCI or PD dementia^28,29(p)^. In contrast to NFL, which relates to axonal damage, disease-relevant pathological accumulations can now be detected at very low concentrations in the plasma. Plasma levels of phosphorylated tau at threonine 181 (p-tau181) is now established in Alzheimer’s and other dementias as a marker of tau as well as β-amyloid pathology.^30^ It is of relevance in PD, as β-amyloid plaques and tau deposition are seen in over three quarters of patients with PD dementia at post-mortem^31^ and may be more strongly related to rates of progression to dementia than Lewy-related pathology.^31^ In people with PD who progress to dementia, p-tau181 levels were not increased, although in the related condition, dementia with Lewy bodies, higher levels of plasma p-tau181 correlated with greater degree of cognitive decline.^29,32^ The relationship between plasma markers such as NFL and p-tau181, with neuroimaging changes in people with PD, and how these relate to poor clinical outcomes is not yet clear.

Establishing the relationship between neuroimaging and plasma measures in people with PD who go on to develop poor clinical outcomes can provide key insights into the sequence of underlying pathological changes in these patients. Specifically, higher order models of diffusion imaging, and plasma NFL can shed light on the role of axonal damage; whereas plasma p-tau181 provides information about brain levels of tau and beta-amyloid accumulation. At the same time, markers predicting poor outcomes in PD can improve efficiency and stratification for clinical trials, as well as more targeted treatment, as new pathology-specific interventions emerge for neurodegenerative disease.

**Figure 1:**
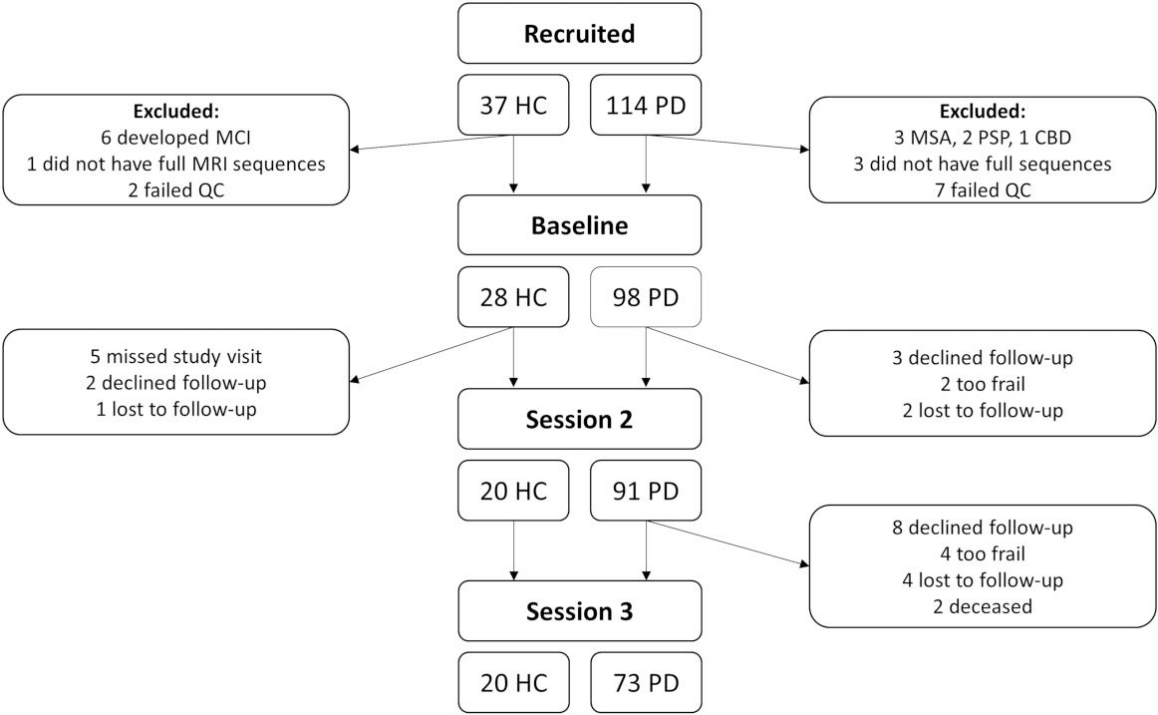
**Overview of the recruited participants.** A total of 114 people with Parkinson’s (PD) and 37 healthy controls (HC) were recruited. After exclusion of PD patients with a change in diagnosis to atypical parkinsonism during the follow-up period, controls who developed mild cognitive impairment (MCI) and those who did not complete the full scanning sequence or failed quality control (QC), a total of 98 patients with PD and 28 HC were included. All participants underwent MRI imaging at baseline (including T_1_-weighted imaging, diffusion weighted imaging and resting state functional MRI). At Session 2, participants had blood taken for plasma markers (neurofilament light and phosphorylated tau). Participants underwent clinical and cognitive assessments at all study sessions. Participants with PD were classified as PD poor outcomes (*n* = 31) if any of the following occurred during follow-up: death, frailty, dementia or Parkinson’s with mild cognitive impairment (PD-MCI). All other Parkinson’s disease participants were classified as Parkinson’s disease good outcomes (*n* = 67). Frailty was defined as the participant becoming too frail or unwell to attend for research sessions by a researcher blinded to imaging and plasma measures. Dementia was defined as a clinical diagnosis of dementia made the treating clinician, or impairment on the functional assessments questionnaire, or MoCA falling below 26 and remaining at <26 at subsequent follow-up. PD-MCI was defined as persistent performance below 1.5 SD in at least two different tests in one cognitive domain or one cognitive test in at least two cognitive domains.^33^ CBD, corticobasal degeneration; MSA, multiple system atrophy; PD-MCI, Parkinson’s with mild cognitive impairment; PSP, progressive supranuclear palsy.

Here, we examined neuroimaging and plasma markers in 98 people with PD followed up over 3 years (Fig. 1). Using multimodal neuroimaging at baseline, we assessed: (i) grey matter changes using cortical thickness; (ii) white matter microstructural and macrostructural changes at whole-brain and tract level using fixel-based analysis, a technique able to reliably model crossing fibres^34^; and (iii) changes in functional and structural connectivity at whole-network level in people with PD who develop poor outcomes during follow-up, defined as the mild cognitive impairment (MCI), dementia, frailty or death, compared to those with PD and who do not develop these poor outcomes. In addition, we assessed the concentration of two plasma biomarkers, NFL and plasma p-tau181, taken during follow-up sessions in people with PD, and related these to neuroimaging measures and to clinical outcomes.

## Materials and methods

### Participants

We recruited 114 people with PD and 37 unaffected controls to the Vision in Parkinson’s disease study (approved by the Queen Square Research Ethics Committee 15.LO.0476) between October 2017 and November 2018. Details on the study protocol have been previously described.^7^ In brief, exclusion criteria were confounding neurological or psychiatric disorders and a pre-existing diagnosis of dementia or MCI. Participants were assessed at baseline, and after 18 (Session 2) and 36 months (Session 3). All patients with PD fulfilled the Movement Disorder Society (MDS) clinical diagnostic criteria.^35^ Six participants were excluded due to their diagnosis being revised after follow-up (two progressive supranuclear palsy, one corticobasal syndrome, three multiple system atrophy), and six control participants due to development of MCI. Four participants did not have full MR sequences available (three PD and one control) and nine failed imaging quality control (seven PD and two controls). This left 98 participants with PD and 28 healthy controls with full baseline clinical and imaging data (Fig. 1).

All participants underwent clinical and neuropsychological assessments at each study session. Assessments were performed with participants receiving their usual medications to minimize discomfort. General measures of cognition included the Mini-Mental State Examination (MMSE)^36^ and Montreal Cognitive Assessment (MoCA).^37^ Additionally, two tests per cognitive domain were performed:^33^ ‘Attention’: digit span^38^ and Stroop colour,^39^ ‘Executive function’: Stroop interference^39^ and category fluency,^40^ ‘Language’: graded naming task^41^ and letter fluency, ‘Memory’: word recognition task^42^ and logical memory^38^ and ‘Visuospatial function’: Benton Judgement of line orientation^43^ and Hooper visual organization test.^44^ A composite cognitive score was calculated as the averaged *Z*-scores of the MoCA plus one task per cognitive domain.^7^ Vision was assessed with LogMAR^45^ for visual acuity, Farnsworth D15^46^ for colour vision and Pelli–Robson^47^ for contrast sensitivity. Mood was assessed using the Hospital Anxiety and Depression Scale (HADS).^48^ All PD participants underwent assessments of motor and non-motor function using MDS-UPDRS^49^ and the Time Up and Go (TUG) test,^50^ sleep using the REM Sleep Behaviour Disorder Questionnaire,^51^ smell using Sniffin’ Sticks,^52^ visual hallucinations using the University of Miami PD hallucinations questionnaire (UMPDHQ)^53^ and number of falls. Levodopa dose equivalence scores (LEDD) were calculated for PD participants.^54^

### Definition of poor outcome

Poor outcome in people with PD was defined as any of the following occurring during follow-up: death, frailty (defined as the participant becoming persistently too frail or unwell to attend for research sessions by a researcher blinded to imaging and plasma measures), dementia (defined as a reported clinical diagnosis of dementia, or impaired functional assessments questionnaire, or MoCA falling below 26 and remaining at <26 at subsequent follow-up)^55^ or PD-MCI (defined as persistent performance below 1.5 SD in at least two different tests in one cognitive domain or one cognitive test in at least two cognitive domains.^33^ Cognitive impairment, frailty and death were chosen as poor outcomes due to their significant functional impact on patients, similar to other longitudinal cohorts.^2,7^ Additionally, we replicated all main analyses comparing only patients with PD who develop dementia or MCI to PD with good outcomes (excluding patients who developed frailty and those who died).

### Data acquisition

All MRI data were acquired on the same 3T Siemens Magnetom Prisma scanner (Siemens) using a 64-channel head coil. Diffusion weighted imaging (DWI) was acquired with the following parameters: b0 (both AP and PA directions), *b* = 50 s/mm^2^/17 directions, *b* = 300 s/mm^2^/8 directions, *b* = 1000 s/mm^2^/64 directions, *b* = 2000 s/mm^2^/64 directions, 2 × 2 × 2 mm isotropic voxels, TE = 3260 ms, TR = 58 ms, 72 slices, acceleration factor = 2, acquisition time ∼10 min. 3D MPRAGE (magnetization prepared rapid acquisition gradient echo) image was acquired with: 1 × 1 × 1 mm isotropic voxels, TE = 3.34 ms, TR = 2530 ms, flip angle = 7°, acquisition time ∼5min. Resting state functional MRI (rsfMRI) was acquired with: gradient-echo EPI, TR = 70 ms, TE = 30 ms, flip angle = 90°, FOV = 192 × 192, voxel size = 3 × 3 × 2.5 mm, 105 volumes, acquisition time ∼7 min. During rsfMRI, participants were instructed to lie quietly with eyes open and avoid falling asleep (confirmed by monitoring and post-scan debriefing). All imaging sequences were performed at the same time of day, with PD participants receiving their normal medications.

### MRI preprocessing and quality assurance

All raw volumes of MPRAGE and DWI images were visually inspected prior to preprocessing and each volume evaluated for the presence of artefact; only scans with <15 volumes containing artefacts^56^ were included. One PD participant was excluded from cortical thickness and structural connectivity analyses for failing quality control of MPRAGE image. Quality of rsfMRI data was assessed using the MRI Quality Control tool.^57^ We excluded participants with: (i) mean framewise displacement (FD) > 0.3 mm; (ii) any FD > 5 mm; or (iii) outliers > 30% of the whole cohort. This led to 12 participants being excluded from rsfMRI sub-analysis (11 PD and 1 control).

Images passing quality assurance underwent preprocessing based on established pipelines from our group.^58,59^ Briefly, FreeSurfer v6.0 was used with default parameters for cross-sectional processing. DWI image preprocessing was performed in MRtrix3.0^60^ and included denoising,^61^ removal of Gibbs artefacts,^62^ eddy-current and motion^63^ and bias field correction.^64^ Images were then up-sampled to 1.3 mm^3^ voxel as recommended for fixel-based analysis,^65^ and intensity normalization was performed across subjects. rsfMRI data underwent standard preprocessing using fMRIPrep 1.5.0^66^: first four volumes discarded; slice-time,^67^ motion^68^ and distortion correction.^69^

Included participant’s MRI images were also assessed using four image quality control metrics: coefficient of joint variation, entropy focus criterion and total signal to noise ratio derived from structural T_1_-weighted images and mean framewise displacement derived from rsfMRI images.

### Fixel-based analysis

From each participant’s preprocessed DWI image, a fibre-orientation distribution was computed using multi-shell three-tissue constrained spherical deconvolution based on the group-average response function for each tissue type (grey matter, white matter, CSF). A group-averaged fibre-orientation distribution template was created from 30 randomly-selected subjects (20 PD, 10 controls). Each participant’s fibre-orientation distribution image was registered to the template^70^ to allow comparisons, and fixel-based metrics were derived: ‘fibre density’: reflecting microstructural changes within tracts, ‘fibre cross-section’: a relative measure of macrostructural changes to an area perpendicular to the white mater fibres and ‘combined measure of fibre density and cross-section (FDC)’: a combined metric of overall tract integrity; FDC is calculated as fibre density multiplied by fibre cross-section for each fixel.^34^

In addition to whole-brain analyses, mean fibre cross-section was calculated within anatomical white matter fibre tracts reconstructed using TractSeg.^71^ The fornix was excluded due to CSF-partial volume effects, and striatal projections were excluded due to their high overlap with obtained thalamic projections. This resulted in 52 white matter tracts included: bilateral arcuate fasciculus, uncinate fasciculus, inferior fronto-occipital fasciculus, middle longitudinal fasciculus, inferior longitudinal fasciculus, superior longitudinal fasciculus I to III, thalamo-prefrontal, thalamo-premotor, thalamo-precentral, thalamo-postcentral, thalamo-parietal, thalamo-occipital, anterior thalamic radiation, superior thalamic radiation, optic radiation, fronto-pontine tract, corticospinal tract, parieto-occipital pontine and inferior cerebellar peduncle, as well as corpus callosum I to VII, anterior commissure, cingulum and middle cerebellar peduncle.

### Connectome construction

Each participant’s T_1_-weighted images was segmented into 360 cortical regions of interest (ROIs) using the Glasser parcellation^72^ and 19 subcortical ROIs from the built-in Freesurfer parcellation.^73^ This parcellation was used to construct both functional and structural connectivity matrices for each participant.

For structural connectomes, diffusion tensor metrics were calculated and constrained spherical deconvolution performed.^74^ Raw T_1_-weighted images were registered to the DWI image using NiftyReg^75^ and five-tissue anatomical segmentation performed using 5ttgen. Anatomically constrained tractography was then performed (10 million streamlines, iFOD2, algorithm^76^), and spherical deconvolution informed filtering of tractograms (SIFT2) was applied to reduce biases.^77^ The resulting set of streamlines was used to construct the structural brain network. Connections were weighted by streamline count and a cross-sectional area multiplier^77^ and combined to a 360 × 360 undirected, weighted matrix. This was not thresholded as recommended by the authors of SIFT2.^77^

For functional connectomes, the preprocessed rsfMRI images were co-registered to the corresponding T_1_-weighted image (boundary-based registration, 6 degrees of freedom).^78^ Physiological noise regressors were extracted using CompCor.^79^ Sources of spurious variance were removed through linear regression (six motion parameters, mean signal from white matter and cerebrospinal fluid), followed by calculation of bivariate correlations and application of Fisher transform. Functional connectivity between ROIs was defined as the Pearson correlation coefficient between mean regional BOLD time series; resulting to a 360 × 360 weighted adjacency matrix representing the functional connectome of each participant.

### Plasma biomarkers

A total of 87 participants had samples taken for p-tau181 and 88 for NFL. Due to COVID-19 lockdown restrictions taking place during part of our Session 2 testing period, 13 of the patients did not undergo plasma sampling at Session 2, and instead underwent sampling in Session 3. The majority of patients underwent testing at Session 2 with 74 patients who had samples taken for p-tau181 and 75 for NFL. p-tau181 concentration was measured using the Simoa P-tau181 Advantage Kit, and NFL concentration was measured using the Simoa Human Neurology 4-Plex A (N4PA) assay (Quanterix). The measurements were performed in two rounds using two batches of reagents with the analysts blinded to diagnosis and clinical data. There was no significant correlation between NFL plasma levels and batch (*β* = 2.503, *P* = 0.119), but there was a batch effect for p-tau181 levels (*β* = 0.901, *P* = 0.001) therefore we corrected for batch number for all analysis of plasma p-tau181 but not NFL levels. All measurements were above the limit of detection of the assays. Only samples with intra-assay coefficients of variation below 10% were included. Plasma data were then matched to phenotype data.

### Statistical analyses

#### Demographics and clinical assessments

At baseline, between-group comparisons were performed using ANOVA for normally distributed continuous variables and Kruskal–Wallis for non-normally distributed ones, with *post hoc* Tukey and Dunn tests, respectively. Visual inspection and the Shapiro–Wilk’s test were used to assess normality. Repeated measures ANOVA were used to assess group differences of cognitive and other clinical assessments longitudinally. Between-group comparisons of plasma biomarkers (PD good versus PD poor outcome) were performed using a general linear model with age at baseline and sex as nuisance covariates. Spearman correlation coefficient was used to assess the relationship between mean fibre cross-section and plasma biomarkers.

##### Whole-brain fixel-based analysis

Connectivity-based fixel enhancement and non-parametric permutation testing were used to identify whole-brain differences in fixel-based metrics as implemented in Mrtrix.^80^ Whole-brain probabilistic tractography was performed on the population template with 20 million streamlines and filtered to 2 million streamlines using spherical deconvolution informed filtering of tractograms (SIFT).^81^ Connectivity-based fixel enhancement was then performed across all white-matter voxels (using the John Hopkins University white matter atlas) with default parameters, 5000 permutations and family-wise error (FWE) correction for multiple comparisons. Statistical significance was set at FWE-corrected *P* < 0.05 with extent-based threshold of 10 voxels. Comparisons between PD good outcome and PD poor outcome were performed with age at baseline, sex and total intracranial volume as nuisance covariates for fibre cross-section and FDC; for fibre density, we did not include total intracranial volume as recommended.^82^ Additional comparisons between PD and control participants, correcting for age, gender and total intracranial volume, were also performed (with the latter not included as a covariate for fibre density analyses, as above). Finally, we performed a conventional voxel-based diffusion tensor imaging analysis using threshold-free cluster enhancement with default parameters (dh = 0.1, E = 0.5, H = 2) across the whole white matter as in the fixel-based analysis, for the same comparator groups, FWE correction *P* < 0.05.

##### Tract-of-interest analysis

As well as whole-brain analysis, we also performed between-group comparisons of mean fibre cross-section for tracts of interest in PD, comparing patients with good versus poor outcomes to validate results. Analyses were performed using a general linear model, with age at baseline, sex and total intracranial volume as nuisance covariates. FDR correction was performed across the 52 included tracts. Additional, longitudinal correlations between tract mean fibre cross-section and change in cognitive scores were performed using linear mixed effects models (implemented in lmer4) with age at baseline, sex, total intracranial volume and time-to-follow-up as covariates. FDR correction was performed across the 10 selected tracts that showed significantly relationships on group comparisons (between PD good versus PD poor outcome).

##### Whole-brain cortical thickness analysis

A general linear model, implemented in Freesurfer, was used to assess differences in cortical thickness at baseline between people with PD with good versus poor outcomes, with cortical thickness as the dependent variable and age at baseline, sex, group (PD good outcome versus PD poor outcome) and total intracranial volume as nuisance covariates. Significance maps were corrected for multiple comparisons using an FDR correction combined over the left and right hemispheres.

#### Grey matter region of interest analysis

To confirm results of the whole-brain cortical thickness analysis and include subcortical grey matter, we performed an additional region of interest analysis between PD poor outcomes and PD good outcomes comparing mean grey matter volume across the 360 regions of the Glasser parcellation^72^ and subcortical regions of the built-in Freesurfer parcellation between the two groups, with age, gender and total intracranial volume as nuisance covariates, and FDR correction combined over the left and right hemispheres.

#### Connectome analysis

Network-based statistics (NBS)^83^ was used to assess differences in structural and functional networks between PD with good outcome and PD with poor outcome, with age and sex as nuisance covariates. Non-parametric permutation testing with unpaired *t*-tests was performed with 5000 permutations calculating a *t*-test for each connection. A threshold of *t* = 3.0 and FWE correction of *P* < 0.05 was applied. Significant networks were visualized using BrainNet Viewer.

## Results

A total of 98 people with PD and 28 healthy controls were included at baseline (Fig. 1). PD participants were defined as having poor outcomes (PD poor outcomes, *n* = 31) if they developed frailty, dementia or MCI at any point during follow-up, or if they died (see ‘Materials and methods’: ‘Participants’ for details). Of those with poor outcomes: 2 died, 6 developed frailty, 11 developed dementia (1 of those also developed frailty and 1 of those died) and 14 developed MCI. All remaining participants were defined as PD good outcomes, *n* = 67.

Demographics and baseline clinical assessments are seen in Table 1. Importantly, the groups did not significantly differ in terms of MRI image quality metrics or medical comorbidities (Supplementary Fig. 1 and Supplementary Table 1). People with PD and poor outcomes were older at baseline (mean ± std: 68.5 ± 8.5 years versus 62.4 ± 7.0 for PD good outcomes), with higher male predominance (74.2% for PD poor outcomes versus 43.3% in PD good outcomes). They also had higher depression scores (5.2 ± 3.3 for PD poor outcomes versus 3.4 ± 2.7 for PD good outcomes), albeit below the clinical threshold ≤ 8. They also performed worse than people with PD and good outcomes and controls in visual assessments including colour (*P* = 0.016) and contrast sensitivity (*P* = 0.006) and in cognitive testing including MMSE (*P* = 0.004), MoCA (*P* < 0.001), Stroop colour (*P* = 0.002), Stroop interference (*P* = 0.001), verbal fluency category (*P* = 0.003), delayed logical memory (*P* < 0.001), Judgement of line orientation (*P* = 0.042) and Hooper visual organization task (*P* < 0.001). Finally, people with PD and poor outcomes had higher baseline total MDS-UPDRS (*P* = 0.007) and MDS-UPDRS motor scores (*P* = 0.042) than people with PD and good outcomes (Table 1). Longitudinal change in cognition and disease-specific measures are presented in Table 2.

**Table 1:** Baseline demographics and clinical assessments

	Healthy controls *n* = 28	PD with good outcome *n* = 67	PD with bad outcome *n* = 31	Statistic *P*-value
Age	**65.7 (9.1)**	**62.4 (7.0)**	**68.5 (8.5)**	**H = 11.280 *P* = 0.004** ^a^
Sex (F/M)	**15/13**	**38/29**	**8/23**	***x* ^2^** = **0.3091 *P*** = **0.014**^a^
Handedness (R/L)	25/3	63/4	27/4	*x* ^2^ = 3.254 *P* = 0.516
Years in education	17.9 (2.3)	16.9 (2.6)	17.5 (2.9)	H = 1.905 *P* = 0.386
Total intracranial volume	1393.7 (95.5)	1459.7 (135.5)	1468.9 (120.5)	F = 0.037 *P* = 0.036^ns^
Mood (HADS)				
Depression score	**2.1 (1.5)**	**3.4 (2.7)**	**5.2 (3.3)**	**H** = **31.231 *P*** < **0.001**^a,b,c^
Anxiety score	3.6 (3.4)	5.5 (3.6)	6.7 (4.8)	H = 8.390 *P* = 0.015^b,c^
Vision				
Acuity (LogMar best)*	−0.07 (0.3)	−0.00 (0.2)	−0.06 (0.2)	H = 2.194 *P* = 0.334
Contrast sensitivity (Pelli–Robson best)	**1.8 (0.2)**	**1.8 (0.2)**	**1.7 (0.2)**	**H** = **8.289 *P*** = **0.016**^a,c^
Colour (D15 total error score)*	**1.2 (0.9)**	**1.1 (0.6)**	**1.9 (1.9)**	**H** = **10.287 *P*** = **0.006**^a,c^
*Cognitive measures*				
Global				
MMSE	**29.2 (0.9)**	**29.1 (1.1)**	**28.4 (1.5)**	**H** = **10.983 *P*** = **0.004**^a,c^
MoCA	**28.9 (1.3)**	**28.5 (1.4)**	**26.3 (3.2)**	**H** = **10.983 *P*** < **0.001**^a,c^
Combined cognitive score	**0.07 (0.6)**	**−0.01 (0.6)**	**−0.93 (0.9)**	**H** = **26.951 *P*** < **0.001**^a,c^
Attention				
Digit span backwards	7.4 (2.0)	7.6 (2.2)	6.3 (2.1)	H = 5.362 *P* = 0.068
Stroop colour naming time (s)*	**31.5 (6.9)**	**32.5 (6.2)**	**39.3 (10.1)**	**H** = **12.856 *P*** = **0.002**^a,c^
Executive function				
Stroop interference reading time (s)*	**53.8 (10.8)**	**57.8 (12.8)**	**74.9 (28.4)**	**H** = **17.026 *P*** < **0.001**^a,c^
Verbal fluency category	**23.3 (4.9)**	**22.8 (5.0)**	**19.0 (6.5)**	**F** = **0.078 *P*** = **0.003**^a,c^
Language				
Graded naming task	23.9 (4.8)	23.9 (2.9)	22.8 (3.5)	H = 6.765 *P* = 0.061
Verbal fluency letter	17.5 (5.7)	17.6 (4.9)	15.0 (6.4)	F = 0.023 *P* = 0.088
Memory				
Word recognition task	24.5 (1.0)	24.3 (0.9)	23.7 (3.5)	H = 6.765 *P* = 0.034^c^
Logical memory (delayed)	**14.6 (3.9)**	**14.4 (3.9)**	**10.5 (4.7)**	**F** = **0.141 *P*** < **0.001**^a,c^
Visuospatial				
Judgement of line orientation	**24.9 (5.9)**	**25.2 (3.8)**	**23.2 (4.1)**	**H** = **6.324 *P*** = **0.042**^a,c^
Hooper	**24.9 (2.1)**	**25.2 (3.0)**	**22.1 (3.1)**	**H** = **28.254 *P*** < **0.001**^a,c^
Disease-specific metrics				
Disease duration		4.1 (2.3)	4.9 (3.4)	U = 993 *P* = 0.209
Affected side at onset (R/L/BL)		35/6/26	14/7/10	*x* ^2^ = 8.264 *P* = 0.082
UPDRS total score		**41.4 (18.7)**	**55.6 (32.9)**	***t*** = **−2.755 *P*** = **0.007**
UPDRS motor score		**19.5 (9.5)**	**26.6 (18.8)**	***t*** = **−2.074 *P*** = **0.042**
LEDD		451.8 (271.4)	484.3 (231.7)	U = 908 *P* = 0.195
Hallucinations (UMPDHQ)		0.7 (1.9)	1.1 (2.2)	U = 953.5 *P* = 0.164
Sleep (RBDSQ)		4.1 (2.5)	4.5 (2.5)	U = 908 *P* = 0.224
Smell (Sniffin’ sticks)		7.9 (3.0)	7.1 (3.3)	*t* = 1.215 *P* = 0.224

All results are shown as mean (standard deviation) unless otherwise specified.

PD, Parkinson’s disease; HC, healthy controls; F, female; M, male; HADS, Hospital Anxiety and Depression Scale; R, right; L, left; BL, bilateral; MMSE, Mini-Mental State Examination; MoCA, Montreal Cognitive Assessment; combined cognitive score, calculated as the mean of the *Z*-scores of 2 cognitive scores per cognitive domain (*Z*-scored against control performance at baseline); UPDRS, Unified Parkinson’s Disease Rating Scale; LEDD, total equivalent levodopa dose; UMPDHQ, University of Miami Parkinson’s Disease hallucinations questionnaire; RBDSQ, REM Sleep Behaviour Disorder Questionnaire. * Lower scores are better.

^a^ *Post hoc* significant difference between PD good and PD poor outcome (also in bold).

^b^ *Post hoc* significant difference between HC and PD good outcome.

^c^ *Post hoc* significant difference between HC and PD poor outcome.

**Table 2:** Longitudinal change in cognitive and disease-specific metrics in patients with Parkinson’s disease (PD)

	Baseline			Session 2			Session 3		
	PD good outcome *n* = 67	PD poor outcome *n* = 31	*P*-value	PD good outcome *n* = 62	PD poor outcome *n* = 29	*P*-value	PD good outcome *n* = 53	PD poor outcome *n* = 20	*P*-value
*Cognitive measures*									
Global									
MMSE	**29.1** (**1.1**)	**28.4** (**1.5**)	**0.002**	28.5 (3.9)	24.7 (10.1)	0.061	**29.2** (**1.2**)	**28.4** (**1.5**)	**0.030**
MoCA	**28.5** (**1.4**)	**26.3** (**3.2**)	**<0.001**	27.9 (2.3)	26.8 (2.7)	0.067	**28.9** (**0.7**)	**26.2** (**2.9**)	**<0.001**
Combined cognitive score	−**0.01** (**0.6**)	−**0.93** (**0.9**)	**<0.001**	**−**0.36 (0.9)	−0.51 (1.1)	0.883	−**0.07** (**0.5**)	**−1.19** (**0.9**)	**<0.001**
Attention									
Digit span backwards	7.6 (2.2)	6.3 (2.1)	0.068	7.7 (2.9)	6.7 (3.3)	0.101	**8.2** (**2.3**)	**7.0** (**2.5**)	**0.031**
Stroop colour naming time (s)	**32.5** (**6.2**)	**39.3** (**10.1**)	**0002**	35.1 (9.3)	36.0 (13.9)	0.626	**32.2** (**5.5**)	**39.2** (**13.3**)	**0.041**
Executive function									
Stroop interference reading time (s)	**57.8** (**12.8**)	**74.9** (**28.4**)	**<0.001**	66.0 (23.9)	61.5 (26.4)	0.111	**54.7** (**15.5**)	**74.3** (**26.6**)	**0.023**
Verbal fluency category	**22.8** (**5.0**)	**19.0** (**6.5**)	**0.044**	19.9 (4.9)	19.7 (4.8)	0.911	**21.1** (**4.4**)	**15.1** (**5.9**)	**<0.001**
Language									
Graded naming task	23.9 (2.9)	22.8 (3.5)	0.061	24.5 (2.9)	23.8 (3.4)	0.838	**25.4**	**20.9**	**0.003**
Verbal fluency letter	17.6 (4.9)	15.0 (6.4)	0.088	16.4 (5.5)	15.4 (5.5)	0.496	17.5 (4.4)	15.3 (5.5)	0.147
Memory									
Word recognition task	24.3 (0.9)	23.7 (3.5)	0.064	24.3 (1.1)	23.8 (1.8)	0.095	24.7 (0.7)	24.1 (1.5)	0.062
Logical memory (delayed)	**14.4** (**3.9**)	**10.5** (**4.7**)	**0.001**	11.3 (4.7)	10.4 (5.6)	0.384	**15.8** (**2.8**)	**10.8** (**3.8**)	**0.003**
Visuospatial									
Judgement of line orientation	**25.2** (**3.8**)	**23.2** (**4.1**)	**0.028**	22.9 (7.3)	21.2 (9.4)	0.243	25.3 (3.8)	20.7 (4.5)	0.077
Hooper	**25.2** (**3.0**)	**22.1** (**3.1**)	**<0.001**	24.5 (3.8)	25.1 (2.9)	0.242	**25.9** (**3.2**)	**20.7** (**3.2**)	**<0.001**
Disease-specific metrics									
UPDRS total score	**41.4** (**18.7**)	**55.6** (**32.9**)	**0.007**	39.7 (22.9)	37.0 (28.6)	0.769	**50.2** (**11.1**)	**63.1** (**12.5**)	**0.008**
UPDRS motor score	**19.5** (**9.5**)	**26.6** (**18.8**)	**0.042**	19.2 (11.4)	19.1 (14.4)	0.948	27.2 (7.9)	31.8 (9.7)	0.064
UMPDHQ	0.7 (1.9)	1.1 (2.2)	0.164	0.9 (2.3)	0.8 (2.0)	0.901	0.8 (2.1)	1.4 (2.8)	0.466
TUG test				10.7 (12.5)	8.8 (3.4)	0.695	9.2 (2.7)	10.8 (4.3)	0.107
Functional activities questionnaire				2.6 (3.9)	1.3 (2.7)	0.193	**1.9** (**2.1**)	**5.5** (**5.3**)	**0.050**
Falls in the last week				**0.0** (**0.2**)	**0.3** (**0.7**)	**0.010**	0.0 (0.1)	0.3 (0.6)	0.078
Falls in the last month				0.1 (0.5)	0.3 (0.9)	0.350	0.1 (0.4)	0.7 (1.7)	0.142
Falls in the last 3 months				0.3 (1.4)	0.4 (1.3)	0.472	0.4 (1.7)	1.9 (4.9)	0.201
Falls in the last 12 months				1.7 (6.8)	0.8 (2.4)	0.267	1.7 (5.9)	2.4 (5.2)	0.666

All results are shown as mean (standard deviation) unless otherwise specified. *P*-values are *post hoc* tests for Group (PD with good versus PD with poor outcomes) at baseline and the interaction between Group (PD with good versus PD with poor outcomes) and Session longitudinally. Significant differences are shown in bold. Information on functional activities and falls was not collected at the baseline visit.

MMSE, Mini-Mental State Examination; MoCA, Montreal Cognitive Assessment; combined cognitive score, calculated as the mean of the *Z*-scores of 2 cognitive scores per cognitive domain (*Z*-scored against control performance at baseline); UPDRS, Unified Parkinson’s Disease Rating Scale; UMPDHQ, University of Miami Parkinson’s Disease hallucinations questionnaire; TUG, time up and go.

### Extensive macrostructural white matter changes at baseline in PD with subsequent poor outcome, in the absence of grey matter changes

No significant differences were seen in cortical thickness at whole-brain level, grey matter volume in a region of interest analysis (adjusting for age, sex and total intracranial volume, FDR-corrected), fractional anisotropy or mean diffusivity between people with PD and poor versus good clinical outcomes at baseline.

**Figure 2:**
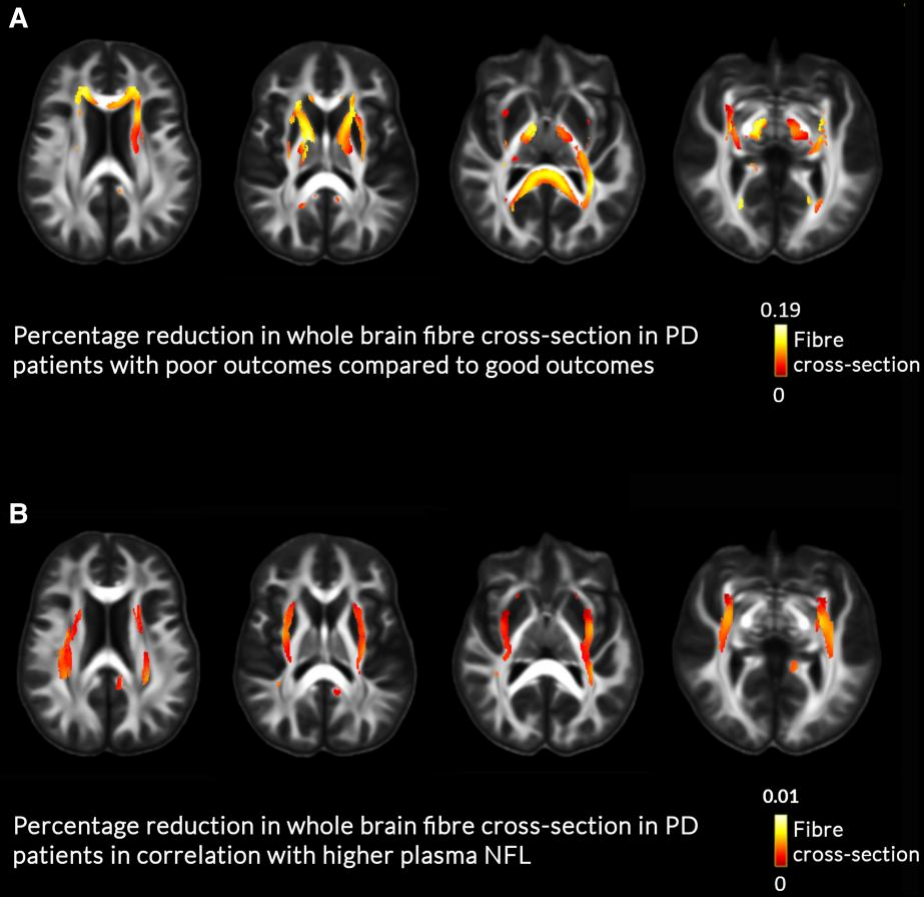
**White matter macrostructural changes in patients with Parkinson’s disease (PD) and poor outcomes.** (**A**) White matter macrostructural changes (percentage reduction in fibre cross-section in PD patients with poor outcomes compared to PD patients with good outcomes at baseline. Whole-white-matter analysis with age, sex and total intracranial volume as nuisance covariates. Effect size is shown as percentage (0–19% reduction), presented as streamlines, only family-wise error (FWE) corrected results are displayed (FWE-corrected *P* < 0.05). (**B**) Reductions in fibre cross-section in PD patients in relation to higher plasma NFL values. Whole-white-matter analysis with age, sex and total intracranial volume as nuisance covariates. The bar shows effect size as percentage (0–1% reduction), presented as streamlines, only family-wise error (FWE) corrected results are displayed (FWE-corrected *P* < 0.05).

We then assessed white matter microstructure (fibre density), macrostructure (fibre cross-section) and overall white matter integrity (combined fibre density and cross-section, FDC) at baseline at whole-white-matter level in people with PD and poor versus good outcomes, adjusting for age, sex and total intracranial volume (for fibre cross-section and FDC). We found extensive macrostructural changes at baseline with up to 19% reductions in people with PD and poor outcomes compared to those with good outcomes in several tracts: bilateral anterior thalamic radiations, optic radiations, inferior fronto-occipital fasciculi, cingulum, thalamo-prefrontal and thalamo-parietal tracts, left corticospinal tract, left middle longitudinal fasciculus, left superior thalamic radiation, left thalamo-parietal tract, left, corpus callosum and middle cerebellar peduncle (Fig. 2A). In contrast, white matter microstructure (fibre density) and FDC did not significantly differ amongst people with PD. Mean fibre cross-section in these areas was correlated with baseline cognition (combined cognitive score at baseline: *r* = 0.228, *P* = 0.026) but not motor scores (MDS-UPDRS part 3: *r* = −0.009, *P* = 0.933). No significant differences were seen in whole-brain analyses between people with PD and controls. In a subgroup analysis examining just PD-MCI/dementia compared with PD good outcomes, we had similar findings with reduction in fibre cross-section in PD patients who developed dementia and MCI compared to those with PD and good outcomes (Supplementary Fig. 3).

**Figure 3:**
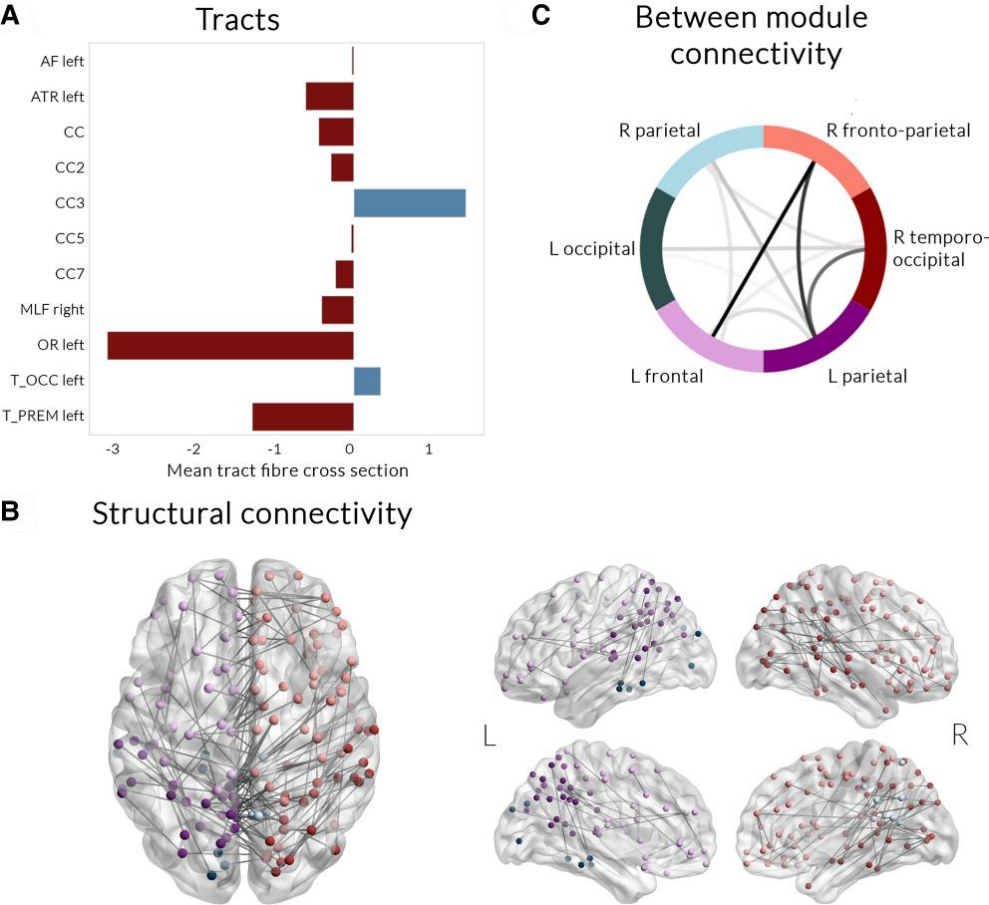
**Changes in tract macrostructure and structural connectivity in patients with Parkinson’s disease (PD) and poor outcomes.** (**A**) Tract-of-interest analysis. Mean fibre cross-section at baseline along 52 white matter tracts, segmented using TractSeg, was compared between PD with poor outcomes versus PD with good outcomes, correcting for age, sex and total intracranial volume, false discovery rate (FDR) corrected for multiple comparisons. PD with poor outcomes showed reduction in mean fibre cross-section in the left arcuate fasciculus (AF), left anterior thalamic radiation (ATR), corpus callosum (CC), specifically the genu (CC2), posterior midbody (CC5) and splenium (CC7), the right medial longitudinal fasciculus (MLF), left optic radiation (OR) and left thalamo-prefrontal tract (T_PREM). Increased mean tract fibre cross-section was seen in PD with poor outcomes in the rostral body of the corpus callosum (CC3) and the left thalamo-occipital tract (T_OCC). All results presented are FDR-corrected *P* < 0.05, presented as percentage change from PD with good outcomes. (**B**) Structural connectivity changes. Network-based statistical analysis revealed a network of reduced connectivity strength in PD with poor outcomes (FDR-corrected *P* < 0.05, *t* = 3.0, 5000 permutations, correcting for age and sex), which comprised 215 edges and 105 nodes across six modules. The subnetwork was visualized using BrainNetViewer with different colours for each module. (**C**) Between modules connectivity changes. The network of reduced structural connectivity in PD with poor outcomes comprised of six modules: R parietal, R frontoparietal, R temporo-occipital, L parietal, L frontal and L occipital. The sum number of connections between modules showing reduced connectivity strength is visualized with darker colour. Connection within R frontoparietal and L frontal, R frontoparietal and L parietal and R temporo-occipital and L parietal modules were most affected in PD with poor outcomes. L, left; R, right.

We then assessed mean fibre cross-section in 52 white matter tracts using TractSeg between people with PD with poor versus good outcomes, adjusting for age, sex and total intracranial volume. We found reduced mean fibre cross-section in PD with poor outcomes within the left arcuate fasciculus, left anterior thalamic radiation, right medial longitudinal fasciculus, left optic radiation and left thalamo-prefrontal tract. Reduced mean fibre cross-section was also seen within the corpus callosum, driven by reductions within the genu, posterior midbody and splenium, whilst increased mean fibre cross-section was seen within the rostral body of the corpus callosum and the left thalamo-occipital tract (Fig. 3A). Results for all 52 tracts included in the analyses are presented in Supplementary Fig. 2. All of the tracts that were significantly different in people with PD with poor outcomes were significantly correlated with combined cognitive scores at baseline (FDR-corrected, adjusting for age, sex and total intracranial volume), and all tracts that showed reduced mean fibre cross-section in PD with poor outcomes were significantly correlated with combined cognitive scores at last follow-up (FDR-corrected, adjusting for age, sex, total intracranial volume and time-to-follow-up) (Table 3). Mean fibre cross-section within the genu of the corpus callosum and the left anterior thalamic radiation were correlated with additional longitudinal change in combined cognitive scores, but neither survived correction for multiple comparisons (Table 3).

**Table 3:** Correlation between tracts of interest showing reduced mean fibre cross-section in PD patients with poor outcomes and cognition, at baseline and longitudinally

Tract	Baseline combined cognitive scores			Longitudinal change in combined cognitive score		
	Coefficient	*P*-value	*q*-value	Coefficient	*P*-value	*q*-value
Arcuate fasciculus left	**0.069**	**0.004**	**0.006**	0.173	0.791	0.857
Anterior thalamic radiation left	**0.101**	**<0.001**	**<0.001**	1.329	0.033	0.165
Genu of the corpus callosum	**0.084**	**<0.001**	**0.001**	1.626	0.019	0.165
Rostral body of the corpus callosum (premotor)	**0.059**	**0.013**	**0.014**	0.122	0.857	0.857
Posterior midbody of the corpus callosum (primary somatosensory)	**0.066**	**0.006**	**0.008**	0.531	0.419	0.698
Splenium of the corpus callosum	**0.090**	**<0.001**	**0.001**	0.88	0.157	0.393
Medial longitudinal fasciculus right	**0.067**	**0.001**	**0.003**	0.459	0.539	0.715
Optic radiation left	**0.079**	**0.002**	**0.003**	0.828	0.151	0.393
Thalamo-occipital tract left	**0.074**	**0.003**	**0.006**	0.73	0.205	0.41
Thalamo-premotor tract left	**0.046**	**0.015**	**0.015**	0.486	0.572	0.715

Correlations were performed between mean tract FC for all 10 tracts that were significantly different between patients with Parkinson’s (PD) with poor versus with good outcomes. Correlations with baseline combined cognitive scores were performed using linear mixed models with age, gender and total intracranial volume as covariates. Longitudinal correlations were performed using linear mixed effects models with age, gender, total intracranial volume and time-to-follow-up as covariates. *q*-value: FDR-corrected *P*-value across the 10 assessed tracts. Significant differences are shown in bold. Combined cognitive scores were calculated as the averaged *Z*-scores of the MoCA plus one task per cognitive domain.

### Plasma NFL but not p-tau181 levels are increased in people with PD with poor outcomes and correlate with white matter macrostructure

**Figure 4:**
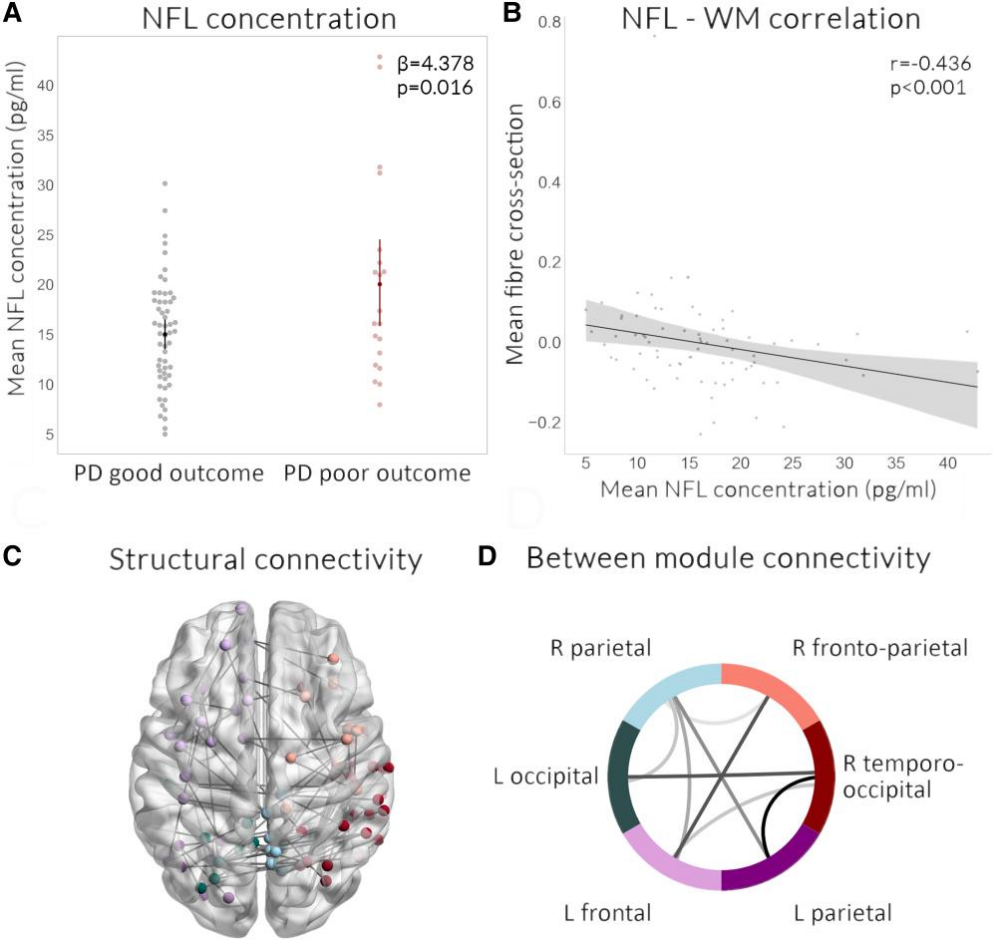
**Changes in plasma NFL in patients with Parkinson’s disease and poor outcomes are associated with white matter changes.** (**A**) NFL concentration. Parkinson’s disease patients with poor outcomes show increased plasma neurofilament light chain (NFL) concentration, adjusting for age and sex, compared to Parkinson’s disease with good outcomes. A general linear model was used with baseline age and sex as nuisance covariates. (**B**) NFL–fibre cross-section correlation. Mean fibre cross-section in areas showing significant whole-white-matter reductions in PD with poor outcomes was significantly correlated (Spearman correlation coefficient) with plasma NFL concentration within patients with PD. (**C**) Structural connectivity changes. Network-based statistical analysis revealed a network of reduced connectivity strength in PD patients in association with higher plasma NFL levels (FDR-corrected *P* < 0.05, *t* = 3.0, 5000 permutations, correcting for age and sex), which comprised 117 edges and 118 nodes across six modules. The subnetwork was visualized using BrainNetViewer with different colours for each module. (**D**) Between modules connectivity changes. Using the same six module allocations of the PD poor outcome network, we visualize with darker colour the sum number of connections between modules showing reduced connectivity strength. Modules included: R parietal, R frontoparietal, R temporo-occipital, L parietal, L frontal and L occipital. L, left; R, right; WM, white matter.

Between people with PD and control participants, plasma NFL did not statistically differ, adjusting for age and sex (*P* = 0.664). However, p-tau181 was higher in people with PD than controls (*β* = 0.555, *P* = 0.038). People with PD with poor outcomes had higher levels of plasma NFL, adjusting for age and sex compared to people with PD with good outcomes (*β* = 4.378, *P* = 0.016), but levels of plasma p-tau181 were not significantly different between groups, adjusting for age, sex and batch (*β* = 0.461, *P* = 0.106) (Fig. 4A and B). This was also found in patients with PD who developed dementia and MCI (Supplementary Fig. 3). Validation of the predictive power of NFL but not p-tau181 in predicting poor outcomes, using k-fold validation, is presented in Supplementary Table 5. Mean fibre cross-section of the areas showing macrostructural changes in people with PD with poor outcomes was significantly negatively correlated with plasma NFL concentration (rho = −0.436, *P* < 0.001) but not p-tau181 levels (rho = −0.153, *P* = 0.157) (Fig. 4B and Supplementary Fig. 4). In keeping with this, on tract-of-interest analyses (corrected for age, sex and FDR-corrected across tracts), higher NFL was associated with lower mean fibre cross-section in four of the tracts showing reductions in people with PD and poor outcomes: left anterior thalamic radiation (*β* = 0.098, *q* = 0.013), right medial longitudinal fasciculus (*β* = 0.072 *q* = 0.001), the genu (*β* = 0.083, *q* < 0.001) and splenium of the corpus callosum (*β* = 0.083, *q* = 0.001). In addition, within people with PD, higher plasma NFL concentration was associated with lower fibre cross-section (up to 1% reductions) at whole-brain analysis within bilateral inferior fronto-occipital fasciculi and optic radiations (Fig. 2B). Mean plasma NFL was correlated with cognition (combined cognitive score) both at baseline (*r* = −0.246, *P* = 0.037) and after 3-year follow-up (*r* = −0.223, *P* = 0.040) as well as motor scores after follow-up (*r* = 0.327, *P* = 0.006) but not at baseline (*r* = 0.027, *P* = 0.804).

### Structural but not functional connectivity is reduced in people with PD with poor outcomes

Finally, we assessed baseline changes at network level in people with PD who had poor outcomes at follow-up, using both functional connectivity from rsfMRI and structural connectivity derived from DWI (age and sex included as nuisance covariates, 5000 permutations, *t* = 3.0, FDR-corrected *P*-value < 0.05). Whist there were no group differences in functional connectivity, we found significant differences in structural connectivity, with reduced connectivity within a network involving 105 nodes and 215 edges and consisting of six modules (*P* = 0.017, Fig. 3B). Connections between right frontoparietal and left frontal, right frontoparietal and left parietal and right temporo-occipital and left parietal modules were most affected in people with PD with poor outcomes (Fig. 3C). A smaller subnetwork also showed a correlation between plasma NFL and poor outcomes in people with PD (*P* = 0.037, 117 nodes, 118 edges, visualized in Fig. 4C and D), but not between p-tau181 and poor outcomes (*P* = 0.994). Significant connections of the network showing reduced connectivity strength in PD with poor outcomes are detailed in Supplementary Table 2; significant connections of the network showing reduced connectivity in correlation with NFL in people with PD are detailed in Supplementary Table 3. Subgroup analysis in patients with PD who developed dementia or MCI is seen in Supplementary Fig. 3 and Supplementary Table 4.

## Discussion

We examined neuroimaging and plasma markers in people with Parkinson’s disease who go on to develop poor clinical outcomes. We show that extensive white matter macrostructural (fibre cross-section) changes can be detected in people with Parkinson’s disease who will progress to poor outcomes, with up to 19% reduction in fibre cross-section affecting multiple tracts, and widespread reductions in structural connectivity. We also found increased levels of plasma markers, particularly NFL in people with Parkinson’s disease who develop poor outcomes. In contrast, cortical thickness, white matter microstructure (fibre density) and functional connectivity are not significantly different in people with PD with poor outcomes compared to those who have good outcomes.

Our findings provide evidence that white matter changes are likely to be important in the pathophysiology of poor outcomes in PD, particularly in relation to cognition, and that white matter imaging is likely to be a more sensitive marker of poor outcomes than grey matter imaging in cognitively intact patients. This is consistent with previous studies assessing whole-brain grey matter volume or cortical thickness in people with Parkinson’s disease who later progressed, which have shown inconsistent findings across regions.^84^ Our negative finding at 3-year follow-up adds further evidence that grey matter methods are poorly sensitive for risk stratification.

We show white matter macrostructural changes (reductions in fibre cross-section) but preserved microstructure (fibre density). Whilst fibre density reflects microscopic changes within intra-axonal volume, fibre cross-section is indicative of macroscopic alterations in a cross-sectional area perpendicular to white matter bundles and is thought to represent the effect of cumulative axonal loss.^34^ Unlike conventional tensor-based metrics, such as fractional anisotropy and mean diffusivity, which aggregate information across multiple fibres within a voxel, fixel-based metrics capture the inherent heterogeneity in white matter organization providing a more nuanced perspective of white matter structure. This is of particular importance in areas of complex crossing fibre architecture, which are abundant in the brain (estimated 60–90% of brain voxels contain crossing fibres^85^) and where tensor-based metrics may give false negative or false positive results.^86^ Several studies (including our own previous work) have shown that fixel-based metrics are more sensitive than conventional metrics in neurodegenerative^22,87,88^ and cerebrovascular disease.^89^ We also found no significant differences in PD patients with poor outcomes in either fractional anisotropy or mean diffusivity, despite the significant widespread changes in fibre cross-section. This provides further support for the use of fibre-specific metrics and particularly fibre cross-section in Parkinson’s.

Interestingly, recent data suggest that white matter macrostructure is specifically affected in the process of neurodegeneration. In a recent study of Alzheimer’s disease, macrostructure (fibre cross-section) was related to pathological beta-amyloid and tau accumulation on PET imaging, whilst microstructural measures such as fibre density were correlated with the presence of white matter hyperintensities,^90^ a measure of small vessel disease. A subsequent study in an independent cohort of MCI patients showed that fibre cross-section was the only fixel-based derived metric associated with higher tau-PET uptake.^91^ Reduced fibre cross-section has also been previously shown in PD compared to healthy controls^22^ and amongst those with poor visual performance who are at a higher risk for dementia.^23^ Although we did not have imaging measures sensitive to small vessel disease in our cohort, our current study provides further evidence of specific macrostructural alterations with preserved microstructure in PD, reflecting neurodegeneration induced axonal loss. Widespread white matter alterations were also seen in our network analysis that revealed extensive structural connectivity changes in PD patients with poor outcomes. Interhemispheric connectivity between frontal, parietal and right occipital regions was most affected, in keeping with the voxel-based fibre cross-section analysis showing particular involvement of the corpus callosum. Prior studies have shown structural connectivity changes in people with established PD-MCI^92,93^ and more recently, people with PD-MCI who later went on to develop PD dementia showed further reductions in both frontal and occipital regions compared with PD patients who did not develop dementia.^20^ Our findings also confirm both frontal and posterior changes in structural intrahemispheric and interhemispheric connectivity, and reveal that these changes are seen in susceptible individuals even before the onset of PD dementia.

Our finding of increased plasma NFL in people with PD and poor outcomes, and correlated with both cognition and follow-up motor scores, is consistent with previous work showing higher CSF NFL concentrations correlated with shorter survival and worse motor symptoms in PD.^94^ Higher plasma NFL in people with PD and established cognitive impairment,^28,29,95^ correlating with rates of cognitive decline,^29^ has also previously been found. Here we now show for the first time, a link between plasma NFL in PD and white matter macrostructural damage, with reduced fibre cross-section within left inferior fronto-occipital fasciculus on whole-brain analysis, and reduced structural connectivity strength involving interhemispheric frontal and parietal connections associated with higher plasma NFL concentration. NFL is a marker of axonal damage,^25,26^ and our findings linking higher levels of plasma NFL with loss of white matter integrity in PD, provide important evidence for the role of axonal damage in Parkinson’s.

Despite extensive changes in structural connectivity, we found no differences in functional connectivity between people with PD with poor versus good outcomes at baseline. There are three possible reasons for this. It could reflect compensatory changes in functional connectivity. In the aging brain, despite an overall reduction in streamlines^96^ and reduced myelin integrity^97^ the functional connectome undergoes extensive reorganization across a posterior–anterior gradient^98^ with both increases and reductions in functional connectivity^98,99^ compensating for the change in structural connectivity and initially preserving cognitive function.^100^ A second reason may be that changes in temporal dynamics rather than static functional connectivity are more sensitive to structural changes, as seen in healthy aging^101^ and in PD-MCI and Parkinson’s dementia.^102^ A third explanation is an effect of levodopa treatment on functional networks. People in our study underwent neuroimaging on their usual dopaminergic medications to limit discomfort with ‘OFF’ effects. Whilst levodopa does not affect structural DWI-derived metrics such as fractional anisotropy,^103^ several studies have shown normalization of functional connectivity changes in people with PD on levodopa compared to controls.^104-106^ In our study, levodopa equivalent doses did not differ between people with PD with poor versus good outcomes, and dopaminergic transmission is less implicated in cognitive impairment in PD^107^ than other neurotransmitters, however normalization of functional connectivity secondary to levodopa may explain the lack of between-group differences in our cohort. Further longitudinal studies of functional connectivity ON and OFF levodopa may clarify whether the lack of static functional connectivity alterations reflects compensatory reorganization or a treatment effect. Longitudinal assessment of functional connectivity incorporating temporal dynamics may be more sensitive as an early marker of poor outcomes in PD.

Similar to other published Parkinson’s cohorts, we did not find a relationship between plasma p-tau181 and cognition^29,95^ or other poor outcomes in PD. Although PD patients had overall higher p-tau181 levels than controls in our cohort, p-tau181 was not correlated with clinical measures nor any of the structural white matter changes we saw in PD with poor outcomes. It is notable that in patients with Dementia with Lewy bodies or at more advanced stages of PD dementia, higher p-tau181 was found to correlate with more rapid cognitive decline.^108^ This could suggest that axonal changes are earlier events in the progression from PD to PD dementia, with pathological accumulation of brain beta-amyloid and tau occurring at later stage. An alternative explanation for our lack of correlation between p-tau181 and PD cognition might be that patients vary in the extent of beta-amyloid and tau accumulation in the brain. Instead of a prognostic biomarker in PD, p-tau181 may have a more useful role in identifying which patients have higher levels of these proteins and could therefore be future candidates for specific anti-amyloid (or anti-tau) therapies.

Finally, our study also highlights demographic and clinical risk factors of poor outcomes, including older age, male gender and poorer visuoperceptual function.^7^ Older age at onset but not disease duration has been highlighted by several epidemiological and pathological studies as a risk factor for poorer clinical outcomes and more rapid rate of progression in PD^109-112^ Beta-amyloid and tau accumulate with aging, even in cognitively intact individuals,^113,114^ as do cerebrovascular disease^115^ inflammation,^116^ impaired autophagy and protein clearance,^117^ mitochondrial dysfunction^118^ and impaired DNA repair.^119^ How these age-related changes interplay with alpha-synuclein and other pathological accumulations in PD will be important to disentangle in future work.

### Limitations and future directions

Our study had some limitations. We included participants within 5 years from diagnosis and patients who subsequently developed poor outcomes. Although they did not fulfil criteria for MCI or dementia, as a group, they showed subtly worse cognitive and motor performance than patients with good outcomes. This is in keeping with other longitudinal cohorts,^2^ but nevertheless, it limits the ability of our study to evaluate the true predictive power of neuroimaging and plasma markers prior to the development of any cognitive signs. Additionally, we followed patients for 3 years, and classified patients as having poor outcomes by the last follow-up session. Inevitably, some patients classified as not having poor outcomes will go on to have poor outcomes with longer follow-up. However, it is important to note that people with PD in our cohort had an average disease duration at baseline of 4.4 years (total disease duration of over 7 years at last follow-up) and 31.6% of PD patients did progress to poor outcomes within this time frame. Indeed in a similar, UK-based prospective study of 142 people with PD with 10-year follow-up, over 60% progressed to dementia, frailty or death within 7 years from diagnosis.^2^ Therefore, our follow-time is sufficient for poor outcomes to occur in a substantial proportion of patients. Nevertheless, longer future studies with longer follow-up in newly diagnosed patients would be ideally suited to assess the value of biomarkers in identifying poor outcomes.

Our MRI sequences did not include T2 or fluid-attenuated inversion recovery sequences, which would be required to quantify concurrent small vessel disease, which is likely to be relevant to outcomes in Parkinson’s,^120,121^ and to understand white matter changes found in our study. Future work could specifically examine this.

Plasma biomarkers were not available in every patient and were only available from follow-up sessions rather than at baseline (with 13 participants only having samples taken at Session 3). This limits the interpretation of these as early markers of poor outcomes in this study. Assays for other phosphorylation targets of p-tau are also emerging: p-tau217 assays have recently been shown to be more sensitive than p-tau181 to PET-amyloid positivity and progression to Alzheimer’s dementia in patients with MCI.^122,123^ Plasma p-tau217 levels are also predictive of abnormal tau-PET and β-amyloid CSF status in dementia with Lewy bodies and PD dementia^124^ but these have not yet been applied in earlier stages of PD and could be examined in future work.

## Conclusions

Here, we examine the changes in neuroimaging and plasma markers in patients with PD who have a poorer clinical outcome at 3-year follow-up. We show extensive macrostructural white matter alterations, with reduction in fibre cross-section, and reduced structural connectivity in interhemispheric frontal, parietal and right occipital connectivity in patients with poor outcomes, in the absence of grey matter or functional connectivity changes. Increased plasma NFL was also found in PD with poor outcomes, correlating with white matter changes. Our study supports the use of white matter macrostructural measures and plasma NFL as markers of poor outcomes before established PD dementia occurs; and provides insights into underlying processes particularly affecting axonal tracts at early stages in the progression to Parkinson’s dementia.

## Supplementary Material

fcae130_Supplementary_Data

## Data Availability

Imaging and clinical data used in this study will be shared upon reasonable request to the corresponding author. Code used in the reported analysis and group-level data can be found here: https://github.com/AngelikaZa/github-AngelikaZa-PoorOutcomeBiomarkersPD.
